# Adaptation of the Spore Discharge Mechanism in the Basidiomycota

**DOI:** 10.1371/journal.pone.0004163

**Published:** 2009-01-08

**Authors:** Jessica L. Stolze-Rybczynski, Yunluan Cui, M. Henry H. Stevens, Diana J. Davis, Mark W. F. Fischer, Nicholas P. Money

**Affiliations:** 1 Department of Botany, Miami University, Oxford, Ohio, United States of America; 2 Department of Chemistry and Physical Science, College of Mount St. Joseph, Cincinnati, Ohio, United States of America; Newcastle University, United Kingdom

## Abstract

**Background:**

Spore discharge in the majority of the 30,000 described species of Basidiomycota is powered by the rapid motion of a fluid droplet, called Buller's drop, over the spore surface. In basidiomycete yeasts, and phytopathogenic rusts and smuts, spores are discharged directly into the airflow around the fungal colony. Maximum discharge distances of 1–2 mm have been reported for these fungi. In mushroom-forming species, however, spores are propelled over much shorter ranges. In gilled mushrooms, for example, discharge distances of <0.1 mm ensure that spores do not collide with opposing gill surfaces. The way in which the range of the mechanism is controlled has not been studied previously.

**Methodology/Principal Findings:**

In this study, we report high-speed video analysis of spore discharge in selected basidiomycetes ranging from yeasts to wood-decay fungi with poroid fruiting bodies. Analysis of these video data and mathematical modeling show that discharge distance is determined by both spore size and the size of the Buller's drop. Furthermore, because the size of Buller's drop is controlled by spore shape, these experiments suggest that seemingly minor changes in spore morphology exert major effects upon discharge distance.

**Conclusions/Significance:**

This biomechanical analysis of spore discharge mechanisms in mushroom-forming fungi and their relatives is the first of its kind and provides a novel view of the incredible variety of spore morphology that has been catalogued by traditional taxonomists for more than 200 years. Rather than representing non-selected variations in micromorphology, the new experiments show that changes in spore architecture have adaptive significance because they control the distance that the spores are shot through air. For this reason, evolutionary modifications to fruiting body architecture, including changes in gill separation and tube diameter in mushrooms, must be tightly linked to alterations in spore morphology.

## Introduction

**Figure 1:**
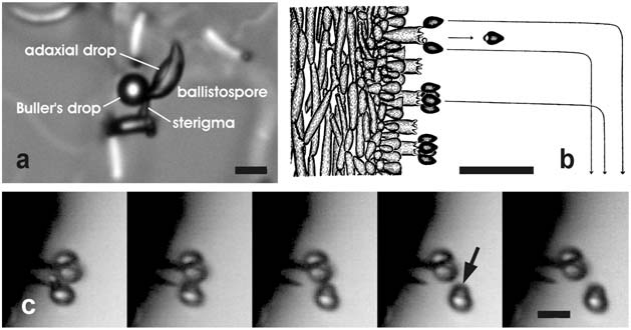
The process of ballistospore discharge. a, Ballistospore of *Tilletia caries* a few seconds before discharge. b, Predicted trajectories of spores discharged from a mushroom gill illustrated by A. H. R. Buller [1]. c, Successive images of ballistospore discharge in *Armillaria tabescens* from video recording obtained at 50,000 fps. Buller's drop (arrowed) carried with discharged spore. Scale bars, a, c, 10 µm b, 50 µm.

The ballistospore discharge mechanism in basidiomycete fungi was first studied by A. H. R. Buller [1], whose name is associated with the fluid drop that forms at the base of the spore a few seconds before discharge (Figs. 1a, S1). Buller's drop is generated by condensation of water from the humid air surrounding the spore [2]. Condensation is driven by the presence of free sugars on the spore surface that lower its water potential [3], [4]. At the same time, water also condenses on the adjacent spore surface and a lens-shaped “adaxial drop” develops. Initially, Buller's drop is separated from the adaxial drop by its position on a knob called the hilar appendix, but the expanding drops coalesce when their surfaces make contact. At the moment of fusion, Buller's drop snaps from the hilar appendix onto the adjacent spore surface. This fluid movement causes a fast redistribution of mass, which imparts momentum to the spore (Fig. 1b,c). Because fluid movement is powered by the reduction in free energy (surface tension) when the two drops fuse, we refer to the mechanism as a surface tension catapult. This mechanism was proposed by Webster and colleagues in 1991 [5], and later verified by high-speed video analysis [6].

**Figure 2:**
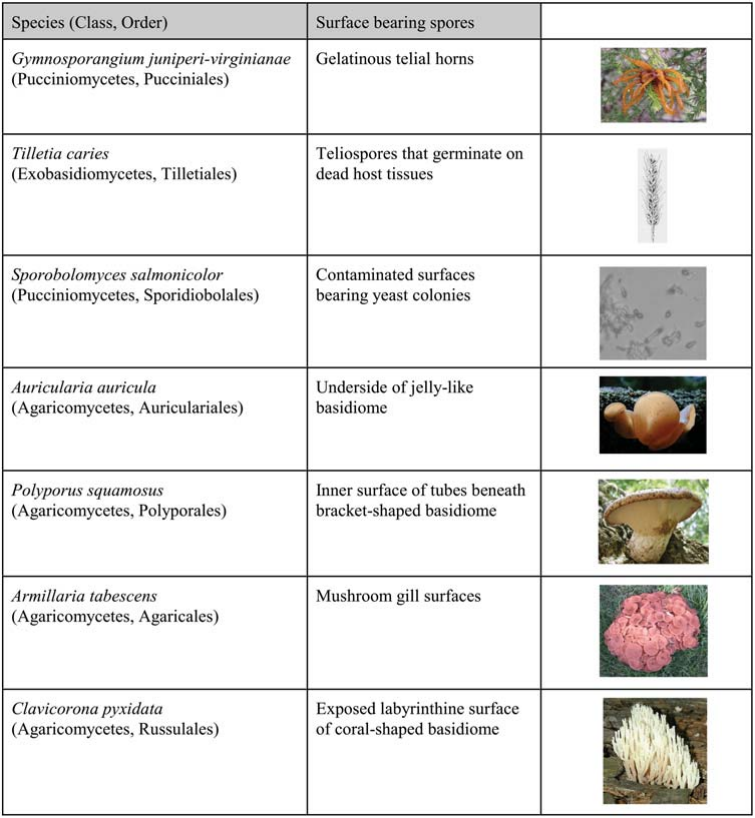
Basidiomycetes selected for this study. Images of *A. auricula* and *C. pyxidata* courtesy of Tom Volk.

Spore discharge involving Buller's drop is characteristic of fungi with an astonishing range of morphology and life history, including unicellular budding yeasts that develop on plant surfaces, mycorrhizal mushrooms, and bracket fungi with enormous fruiting bodies that release trillions of spores [7]. The surface tension catapult is not capable of the discharge distances characteristic of squirt gun mechanisms like the explosive asci of ascomycete fungi and the sporangiophore of *Pilobolus* [8]. Instead, the maximum range of the ballistospore discharge mechanism has been estimated at 1–2 mm in species that discharge their spores from exposed surfaces [9], [10]. These ranges would be catastrophic for mushroom-forming species whose spores are formed on closely-packed gills or within narrow tubes (Fig. 1b). The way in which the range of the mechanism is controlled has not been studied previously, partly because ultra-high-speed video cameras needed to measure discharge velocity have not been widely available until recently. In this paper, we report high-speed video analysis of spore discharge in selected basidiomycetes ranging from yeasts to wood-decay fungi with poroid fruiting bodies (Fig. 2). The species chosen for this study were: *Gymnosporangium juniperi-virginianae*, *Tilletia caries*, *Sporobolomyces salmonicolor*, *Auricularia auricula*, *Polyporus squamosus*, *Armillaria tabescens*, and *Clavicorona pyxidata* [7]. *G. juniperi-virginianae* is a pathogenic fungus that causes cedar-apple rust; its ballistospores are discharged from the surface of orange gelatinous horns that emerge from galls that develop on various juniper species. *T. caries* is another pathogen that causes bunt, or stinking smut, of wheat. *A. auricula* is a saprobe that forms gelatinous fruiting bodies on decaying wood. *S. salmonicolor* is a mirror yeast that thrives in damp conditions; its airborne spores are a common cause of allergy in water-damaged buildings. *P. squamosus*, *A. tabescens*, and *Clavicorona pyxidata* are widespread wood-decay fungi that produce, respectively, large brackets with tubes on their underside, gilled mushrooms, and erect fruiting bodies shaped like coral. Analysis of video data, coupled with mathematical modeling, show how discharge distance is controlled and poses intriguing questions about the evolution of spore shape and size.

## Results and Discussion

**Figure 3:**
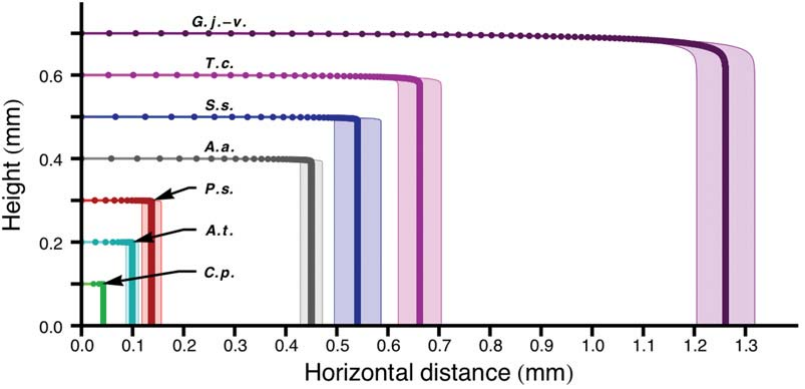
Model spore trajectories for seven basidiomycetes species based on measurements of spore size and launch speed and using Stokes model of viscous drag. To aid visualization, spores were launched horizontally from arbitrary heights. Positions of spores at 50 µs intervals indicated by dots. The variation in horizontal range predicted from the measured variation in launch speeds (±standard error; Table 1) for each species is represented by the shaded region around each trajectory. Species initials: *G.j.-v.*, *Gymnosporangium juniperi-virginianae*; *T.c.*, *Tilletia caries*; *S.s.*, *Sporobolomyces salmonicolor*; *A.a.*, *Auricularia auricula*; *P.s.*, *Polyporus squamosus*; *A.t.*, *Armillaria tabescens*, and *C.p.*, *Clavicorona pyxidata*.

Spore discharge in all of the species in this study is achieved by the formation and collapse of the Buller's drop on the wet spore surface (Fig. 1, Videos S1, S2). Mean launch speeds varied from 0.58 to 1.42 m s^−1^, but the variation in the size of the spores and their Buller's drops produced a 30-fold range in predicted discharge distance, from 0.04 mm in *C. pyxidata* (smallest spore) to 1.26 mm in *G. juniperi-virginiaeae* (largest spore; Fig. 3, Table 1). Acceleration varied from 32,400 m s^−2^ in *A. auricula*, to 140,000 m s^−2^ (14,000 *g*) in *T. caries*. These estimates of acceleration are approximately ten-fold slower than those characteristic of spore launches driven by turgor pressure [8]. Greater discharge distances correlated with faster launch speeds, and the predicted ranges were corroborated by direct measurements of spore ranges from video recordings of discharge in the three species with the smallest spores (Table 1, Fig. 1c). The species with the shorter ranges (≤0.1 mm) propel their spores from fertile tissues supported by macroscopic fruiting bodies. In these fungi, short ranges are likely to limit wastage of spores due to impaction on the inner surface of tubes, opposing gills, or on the convoluted surface of coralloid fruiting bodies. The species that discharged their spores over distances ≥0.5 mm liberate them directly into the airstream, thereby increasing their probability of escaping the boundary layer of sluggish air within which the spores develop. These observations are consistent with our hypothesis that there is adaptive significance to the differences in the range of the discharge mechanism.

**Table 1:** Ballistics of spore discharge in seven basidiomycete species.

Species (Class, Order)	Spore dimensions^1^, length×width	Radius of Buller's drop	Initial velocity, m s^−1^, measured range, mean±s.e.m. (sample size)	Calculated discharge distance^2^, [measured distance]
*Gymnosporangium juniperi-virginianae* (Pucciniomycetes, Pucciniales)	20.0×15.1 µm (19)	5.2 µm (19)	0.66–1.35, 1.11±0.06 (18)	1.26±0.06 mm
*Tilletia caries* (Exobasidiomycetes, Tilletiales)	21.4×7.6 µm (9)	5.2 µm (28)	0.32–1.53, 1.10±0.07 (21)	0.66±0.04 mm
*Sporobolomyces salmonicolor* (Pucciniomycetes, Sporidiobolales)	11.5×7.9 µm (14)	3.8 µm (14)	1.08–1.83, 1.42±0.12 (6)	0.54±0.04 mm
*Auricularia auricula* (Agaricomycetes, Auriculariales)	12.9×7.8 µm (15)	3.1 µm (15)	0.87–1.62, 1.25±0.06 (13)	0.45±0.02 mm
*Polyporus squamosus* (Agaricomycetes, Polyporales)	14.0×5.4 µm (31)	2.6 µm (4)	0.45–0.68, 0.58±0.08 (6)	0.14±0.02 mm [0.13±0.02 mm (6)]
*Armillaria tabescens* (Agaricomycetes, Agaricales)	6.8×6.1 µm (16)	1.5 µm (16)	0.12–0.91, 0.64±0.08 (9)	0.10±0.01 mm [0.06±0.01 mm (4)]
*Clavicorona pyxidata* (Agaricomycetes, Russulales)	4.7×3.5 µm (6)	1.2 µm (6)	0.52–0.87, 0.69±0.06 (5)	0.042±0.004 mm [0.036±0.003 mm (5)]

1 Mean values for spore dimensions and Buller's drop radius, standard errors ≤0.3 µm and ≤0.1 µm, respectively, sample sizes in parenthesis.

2 Distance calculated using mean values for spore dimensions, drop size, and velocity.

**Figure 4:**
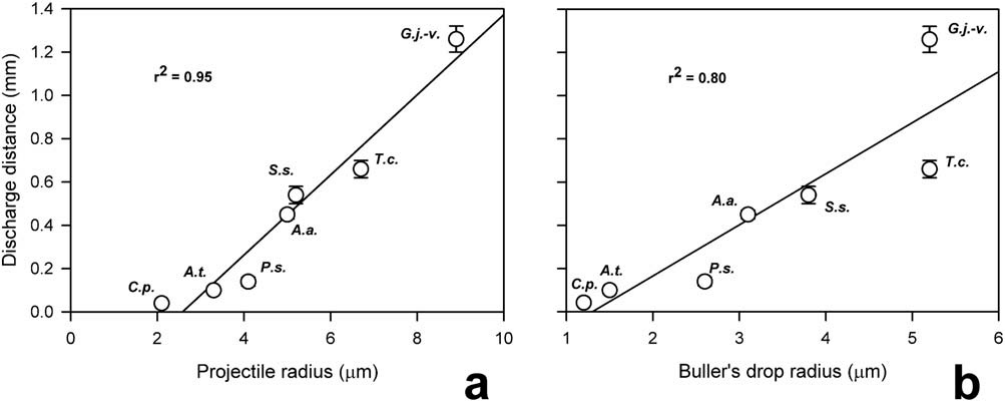
Relationship between discharge distance and (a) projectile radius (spore plus adhering fluid), and (b) Buller's drop radius in the seven basidiomycete species examined using high-speed video. Species initials same as Fig. 3.

Discharge distance was strongly correlated with both projectile size (spore plus adhering fluid) and the size of Buller's drop (Fig. 4). The decelerating drag force is proportional to the square of the spore radius (*r* ^2^), while spore inertia (mass) is proportional to *r* ^3^. Thus, the deceleration (a = F/m) decreases with increasing spore size leading to longer flights. This physical principle is reflected in measurements of discharge distances obtained from 37 mushroom species by Zoberi [10]. The effect of drop size on range is more complicated. Because the drop merges with the spore, its mass also becomes part of the projectile and increases inertia. But because the energy to power the launch is derived from the reduction of surface tension energy as the drop shifts onto the spore surface, the kinetic energy for the launch increases with drop size. Finally, these relationships are complicated by the fact that large spores tend to produce larger drops, and small spores produce smaller drops. Spore and drop size are not independent variables.

The details of drop movement differ from species to species. In *A. tabescens*, for example, the adaxial fluid on the adjacent spore surface does not form a prominent lens shape before discharge, and Buller's drop flips onto the spore and is carried as a discrete hemisphere during the launch (Fig. 1c). In this case, the collapse of the drop is associated with at least a 20% decrease in surface free energy (see Text S1). This means that a maximum energy of 0.4×10^−12^ J is available for the launch, which is ten-fold greater than the kinetic energy of the spore (plus drop) during its launch (½mν^2^ = 3.5×10^−14^ J). The additional energy is probably consumed in severing the connection between the spore and its perch (sterigma). In species with larger spores, like *T. caries* (Fig. 1a), the merger of Buller's drop with a prominent adaxial drop is associated with a much greater change in surface tension that powers the longer flight of the spore.

**Figure 5:**
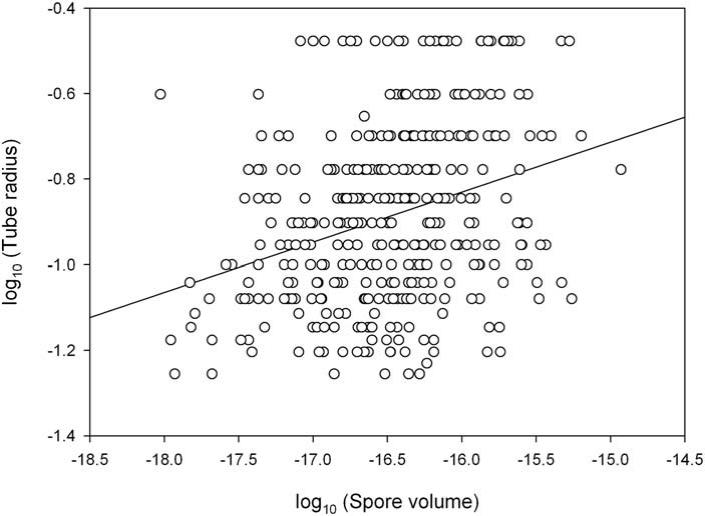
Relationship between spore volume and tube radius. Scatter plot shows data for 382 species of mushroom-forming fungi (Agaricomycetes) with poroid fruiting bodies. The line indicating least squares fit has an *r^2^* value of only 0.098 (P<0.0001), indicating a poor association between the plotted variables.

So how is discharge distance attuned to the diversity of ecological challenges and fruiting body morphology among the 30,000 species of ballistosporic Basidiomycota? The mass of ballistospores varies over three orders of magnitude, from approx. 3 pg (the spores of *C. pyxidata* in our study are approximately ten-fold larger than the smallest ballistospores) to several nanograms (with an estimated mass of 3 ng, the spores of *G. juniperi-virginiae* are among the largest [11]). To examine whether variation in spore size may have served as a major factor in the evolution of discharge distance, we analyzed the association between spore size and tube radius in 382 species of Agaricomycetes with poroid fruiting bodies [12], [13]. The underlying assumption here is that shorter discharge distances are likely to be associated with narrower tubes, but our analysis revealed only a very weak correlation between these variables (Fig. 5). It seems possible, therefore, that changes in the size of Buller's drop have served as a more significant variable in the evolution of discharge distance. Buller's drop forms by condensation of water upon the spore surface, and mannitol and glycerol are the dominant hygroscopic compounds found in washings from the surface of discharged spores in all of the species that we have examined. Differences in osmolyte concentration and relative humidity will effect different rates of drop expansion [3], but the maximum size of the Buller's drop – which determines launch energy – is controlled by spore shape, hydrophobicity of the spore surface, and the size of any adaxial drop. Differences in discharge distance among the basidiomycetes might, therefore, have evolved via adaptive changes in these features of spore architecture. The taxonomic literature on fungi illustrates a tremendous range in spore morphology among the basidiomycete fungi [14]. This variation may have adaptive significance in terms of the operation of the ballistospore discharge mechanism.

## Materials and Methods

### Organisms and culture methods

This study utilized a selection of fresh fruiting bodies, infected plant tissues, and axenically-cultured fungi (Fig. 2). Galls bearing telial horns *Gymnosporangium juniperi-virginianae* were collected from infected eastern red cedar (*Juniperus virginiana*) in Butler County, Ohio. Telial horns were cut from the galls and placed in empty Petri dishes with pads of wet filter paper to maintain humidity. Ballistospores formed on the surface of these horns within a few hours and their discharge was captured using high-speed video microscopy. Some galls were air dried and later rehydrated for study. Teliospores of *Tilletia caries* from infected spring wheat kernels were spread on distilled water agar (DWA), incubated for 5 d at 15 C until primary sporidia developed, then transferred to room temperature for analysis of ballistospore (secondary sporidia) discharge. *Sporobolomyces salmonicolor* strain NPM01 was isolated from a contaminated manufacturing facility. This yeast was cultured on potato dextrose agar and vast numbers of ballistospores formed on the surface of the colonies within 24 h of inoculation. Fresh fruiting bodies (basidiomata) of *Auricularia auricula*, *Polyporus squamosus*, *Armillaria tabescens*, and *Clavicorona pyxidata* were collected from urban gardens and woodland in Oxford, Ohio, and studied within a few hours of harvest. To image spore discharge from these species, thin slices of basidiome (<1 mm) were cut with a scalpel and placed on the surface of DWA in Petri dishes.

### Ultra-high-speed video microscopy

Video recordings were made with FASTCAM-ultima APX and APX-RS cameras (Photron, San Diego, CA) attached to an inverted compound microscope fitted with long-working distance objectives (Olympus, Tokyo). Each video clip was compiled from ≤100 image files extracted from recordings consisting up to 1 million images captured in ≤4 s (e.g., 1 million image files captured with 2 µs shutter at 250,000 fps in 4 s). Analysis of digital images was performed using VideoPoint v.2.5 (Lenox Softworks, Lenox MA), Image-Pro Plus 6.2 (Media Cybernetics, Bethesda, MD), and proprietary software from Photron.

### Analysis of osmolytes on spore surface

Spore deposits were collected from fresh specimens and cultures of the fungi in this study. Petri dishes containing sporulating cells and basidome slices were inverted so that dense deposits of spores collected on lids. The lids were air dried and stored at room temperature for subsequent analysis. Sugars and sugar alcohols contributing to sap osmolality were identified and quantified using GC/MS as described previously [8].

### Mathematical modeling

For spores moving at the speeds observed, viscous forces dominate over inertial forces. Thus, Stokes' law, 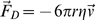, relates the drag force, 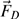, to *r*, the aerodynamic radius of the projectile, 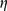, the viscosity of the air, and 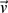, the particle velocity. Analytical integration of Newton's second law, 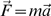, yields expressions for the *x*- and *y*-positions of the spore as functions of time that can be plotted parametrically to determine the spore trajectory. Spore motion in all but one of the species examined in this study (*Gymnosporangium juniperi-virginianae*) fell within the laminar flow regime, characterized by Reynolds numbers below 1.0. A quasi-empirical model for viscous drag has been proposed from particles moving through fluids at the onset of turbulence [15], a regime characterized by Reynolds numbers between 1 and 1,000. Nevertheless, the simpler Stokes model for drag was used for analysis of all of the fungi, because for Reynolds numbers close to 1.0 the difference between the two drag models is slight.

## Supporting Information

Figure S1 Schematic showing process of ballistospore discharge. Buller's drop and adaxial drop form via condensation of water on the spore surface and their coalescence causes a rapid shift in the center of mass of the spore that is responsible for the launch.
(0.44 MB PDF)

Text S1 Energy available for spore discharge.
(0.12 MB DOC)

Video S1 Ultra-high speed video clip showing ballistospore discharge in gilled mushroom of Armillaria tabescens. Frame rate = 50,000 f.p.s.
(3.63 MB AVI)

Video S2 Ultra-high speed video clip showing ballistospore discharge in rust fungus Gymnosporangium juniperi-virginianae. Frame rate = 60,000 f.p.s.
(0.40 MB AVI)
