# Supplementary figures and images for: Adaptation of the Spore Discharge Mechanism in the Basidiomycota
Source: PLoS One. 2009 Jan 8;4(1):e4163. doi: 10.1371/journal.pone.0004163 (PMC2612744; doi:10.1371/journal.pone.0004163)

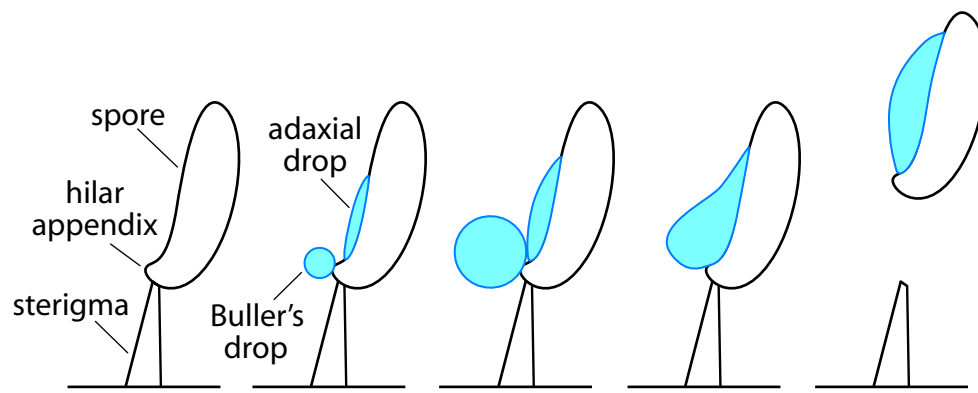

Supplement: Figure S1 — Schematic showing process of ballistospore discharge. Buller's drop and adaxial drop form via condensation of water on the spore surface and their coalescence causes a rapid shift in the center of mass of the spore that is responsible for the launch. (0.44 MB PDF) [file pone.0004163.s001.pdf]
