# Supplementary material for: Adaptation of the Spore Discharge Mechanism in the Basidiomycota
Source: PLoS One. 2009 Jan 8;4(1):e4163. doi: 10.1371/journal.pone.0004163 (PMC2612744; doi:10.1371/journal.pone.0004163)
Supplement: Text S1 — Energy available for spore discharge. (0.12 MB DOC) [file pone.0004163.s002.doc]

**Energy available for spore discharge**

Ballistospore discharge is powered by the motion of Buller’s drop and this is driven by the decrease in surface tension energy density. When Buller’s drop moves onto the spore surface of some species, notably those with the smallest spores in this study, it is often carried with the discharged spore as a discrete hemisphere. If the drop moves onto a dry spore surface, the sphere-to-hemisphere transformation is associated with a 20% change in exposed surface area. If the drop merges with a wet patch on the spore surface (modeled as a disc with negligible volume), then the change in surface area, and energy available for discharge, increases to 60% of the original energy in the Buller’s drop. Finally, the fusion of Buller’s drop with a hemispherical adaxial drop, to form a larger hemispherical drop, is associated with an intermediate change in surface tension energy density of 46%. Here we show the calculation for the most energetically favorable drop movement.

Consider a spherical Buller’s drop merging with a wet patch on the adjacent spore surface to form a hemisphere:

The volume of fluid does not change as the drop is transformed from a spherical to hemispherical shape. Thus,

$$\frac{4}{3}\pi r_{s}^{3}=\frac{1}{2}\left(\frac{4}{3}\pi r_{HS}^{3}\right)$$

From this equation, it follows that:

$$r_{HS}=2^{\frac{1}{3}}r_{s}$$

Upon merger, a surface area the size of the footprint of the hemisphere is lost. Thus, the change in surface area is given by the following expression:

$$\begin{matrix} 4\pi r_{S}^{2}+\pi r_{HS}^{2}-\frac{1}{2}4\pi r_{HS}^{2}=4\pi r_{S}^{2}-\pi r_{HS}^{2} \\ =4\pi r_{S}^{2}\left(1-\frac{1}{4}\left(2^{\frac{1}{3}}\right)^{2}\right) \\ =4\pi r_{S}^{2}\left(1-2^{-\frac{4}{3}}\right) \\ \approx4\pi r_{S}^{2}\left(0.603\right) \end{matrix}$$

Finally, the available energy is:

Some fraction of this total available energy will be converted to kinetic energy of the spore and adhering drop.
